# Comprehensive comparison of in silico MS/MS fragmentation tools of the CASMI contest: database boosting is needed to achieve 93% accuracy

**DOI:** 10.1186/s13321-017-0219-x

**Published:** 2017-05-25

**Authors:** Ivana Blaženović, Tobias Kind, Hrvoje Torbašinović, Slobodan Obrenović, Sajjan S. Mehta, Hiroshi Tsugawa, Tobias Wermuth, Nicolas Schauer, Martina Jahn, Rebekka Biedendieck, Dieter Jahn, Oliver Fiehn

**Affiliations:** 1 Technische Universität Braunschweig - Institute of Microbiology, Brunswick, Germany; 2 Metabolomic Discoveries GmbH, Potsdam, Germany; 3 NIH West Coast Metabolomics Center, UC Davis Genome Center, Room 1313, 451 Health Sci Drive, Davis, CA 95616 USA; 4 Inovatus Ltd, Zagreb, Croatia; 5 RIKEN Center for Sustainable Resource Science, Yokohama, Kanagawa Japan; 6 Department of Biochemistry, Faculty of Sciences, King Abdulaziz University, Jeddah, Saudi Arabia

**Keywords:** Compound identification, Mass spectrometry, Structure elucidation, In silico fragmentation, MS/MS, Metabolomics

## Abstract

In mass spectrometry-based untargeted metabolomics, rarely more than 30% of the compounds are identified. Without the true identity of these molecules it is impossible to draw conclusions about the biological mechanisms, pathway relationships and provenance of compounds. The only way at present to address this discrepancy is to use in silico fragmentation software to identify unknown compounds by comparing and ranking theoretical MS/MS fragmentations from target structures to experimental tandem mass spectra (MS/MS). We compared the performance of four publicly available in silico fragmentation algorithms (MetFragCL, CFM-ID, MAGMa+ and MS-FINDER) that participated in the 2016 CASMI challenge. We found that optimizing the use of metadata, weighting factors and the manner of combining different tools eventually defined the ultimate outcomes of each method. We comprehensively analysed how outcomes of different tools could be combined and reached a final success rate of 93% for the training data, and 87% for the challenge data, using a combination of MAGMa+, CFM-ID and compound importance information along with MS/MS matching. Matching MS/MS spectra against the MS/MS libraries without using any in silico tool yielded 60% correct hits, showing that the use of in silico methods is still important.

**Electronic supplementary material:**

The online version of this article (doi:10.1186/s13321-017-0219-x) contains supplementary material, which is available to authorized users.

## Background

Many fields of research, from environmental analysis to forensics and biology, are moving towards hypothesis-generating screening approaches using liquid chromatography coupled with tandem mass spectrometry (LC–MS/MS) [1, 2]. Such an approach yields hundreds to thousands of signals per study, most of them having unidentified structures even after comprehensive searches of existing mass spectral libraries such as NIST, MassBank, Metlin or MassBank of North America (MoNA). Overall, tandem mass spectral databases cover less than one per cent of the compound space that is covered in Chemspider or PubChem with 50 to 90 million compounds, respectively. As an alternative strategy for compound annotation of known compounds in silico fragmentation software tools have been developed and are used to identify MS/MS spectra when the reference MS/MS spectrum is not available. Such software tools include MetFrag [3], MIDAS [4], MAGMa [5, 6], MAGMa+ [7], MOLGEN–MS/MS [8], CSI:FingerID [9], CFM-ID [10], FingerID [11], Input output kernel regression (IOKR) [12] and the MS-Finder software [13]. A number of commercial software solutions such as MassFrontier (HighChem), MS-Fragmenter (ACD/Labs) or Molecular Structure Correlator (Agilent) are also available, but lack open access code or algorithm transparency.

The data for our investigation was obtained from the CASMI website (http://www.casmi-contest.org/2016/). The Critical Assessment of Small Molecule Identification (CASMI) contest was founded in 2012 to help scientists with their compound identification methods by providing community challenges and competitions [14]. For practical reasons, including the source code and model availability, error handling, batch processing capabilities and the ability to perform local database queries, we only covered in silico fragmentation software that was used for results submitted by the CASMI 2016 deadline.

We surveyed four different tools, all using different algorithms for in silico fragmentation, MetFragCL, CFM-ID, MAGMa+ and MS-FINDER. MetFragCL retrieves candidate structures and fragments them using a bond dissociation approach and those fragments are then compared to the product ions in a measured spectrum to determine which candidate best explains the measured compound by assigning it a score that is a function of the mass to charge ratio (*m/z*), intensity and bond dissociation energy (BDE) of the matched peaks, while 5 of neutral loss rules account for rearrangements [3]. CFM-ID (competitive fragment modelling) employs a method for learning a generative model of collision-induced dissociation fragmentation [10]. CFM-ID can be used to assign fragments to spectra to rank the candidates, but also to predict MS/MS spectra from structures alone. MAGMa+ is a parameter-optimized version of the original MAGMa software [5]. MAGMa analyses substructures and utilizes different bond dissociations. It furthermore calculates a penalty score for all the bonds that are disconnected and form a specific substructure [15]. The improved MAGMa+ version utilized a parameter optimization approach to find optimal processing parameters [7]. The MS-FINDER algorithm simulates the alpha-cleavage of linear chains up to three chemical bonds and considers also bond dissociation energies. Multiple bonds (double, triple, or cycles) are modelled as penalized single bonds in which hydrogens are lost (hydrogen rearrangement rules). The total score also includes mass accuracy, isotopic ratio, product ion assignment, neutral loss assignment and existence of the compound in an internal structure database [13, 16]. First-principle quantum chemical models for spectrum prediction [17] have only been developed for electron ionization but not for electrospray collision-induced dissociation tandem mass spectrometry (ESI-CID-MS/MS).

The CASMI 2016 contest consisted of three categories. Category 1: “Best Structure identification on Natural Products”, with 19 natural product dereplication challenges. The data for Categories 2 and 3 consist of training sets and challenge sets of 312 and 208 of known compounds, respectively. For Category 2: “Best Automatic Structural Identification—In Silico Fragmentation” no other information than the in silico fragmentation was allowed [18]. Category 3: “Best Automatic Structural Identification—Full Information” allowed for any type of additional information to be used, including mixed models, structure rankings and MS/MS search [19].

In order to obtain the ground truth of performance of in silico fragmentation software it is important to exclude all pre-knowledge or any bias such as molecular formula lookup, database ranking or any other means that would influence the score. Furthermore, it is important to include a large number of unknown compounds in order to improve the statistical power of the investigation. We therefore chose the 312 training and 208 challenge MS/MS spectra for investigating the capabilities of current software to perform unbiased batch processing of hundreds of test and validation cases. Additionally, we compare the tools’ performances when more information is allowed to be used and how consensus modelling can improve results.

## Methods

### Tandem mass spectral input data

The CASMI 2016 website (http://www.casmi-contest.org/2016/) provided 312 training and 208 validation files containing MS/MS information as *.MGF file. The MS/MS spectra were acquired on a Q Exactive Plus Hybrid Quadrupole-Orbitrap mass spectrometer (Thermo Fisher), with <5 ppm mass accuracy and MS/MS resolution of 35,000 using ESI ionization. Spectra were collected in stepped 20, 35 and 50 eV in mode. Only [M+H]^+^ (positive) and [M−H]^−^ ion species were available. Spectral meta-data included the ChemSpider ID, compound name, the monoisotopic mass, molecular formula, SMILES, InChI and InChIKey. Some of the candidate structures provided by the organizers were erroneous and did not match the provided formula, SMILES or InChIKey. After the contest deadline, the CASMI organizers provided all correct results for the 312 training and 208 challenge cases that were used in our evaluation.

### Query compounds from ChemSpider

For each of the training and validation cases the CASMI team provided possible candidate lists. These compounds were obtained from ChemSpider with a ±5 ppm search window and the structure files contained the ChemSpider ID, compound name, monoisotopic mass, molecular formula, SMILES, InChI and InChIKey. Because compound masses are unevenly distributed, some mass spectra yielded up to 8000 possible structure candidates within the 5 ppm mass window, whereas one mass spectrum was only associated with a single possible candidate structure, pentabromophenol. A total of 432,511 candidates were available for the training set and 258,808 candidates were obtained for the validation set (challenge set).

Each of the four software tools used different structure-handling libraries or routines, hence structure conversion issues occurred. Such errors can be attributed to salt forms, isotopic elements, radical compounds and conversion issues. Each of the four tested software tools required different input formats and output formats. For that purpose, an application was written in Java to process all output files and analyse the results. It also includes short scripts to help with preparing input files for each tool. Source and result files can be found under (https://sourceforge.net/projects/insilico-fragmentation/).

### Software settings

#### MS-FINDER

The MS-FINDER software (version 1.70) was downloaded from the Riken institute website (http://prime.psc.riken.jp/Metabolomics_Software/MS-FINDER/index.html) and was used on a standard personal computer with a 2.50 GHz Intel Core-i7 CPU and 16 GBytes of RAM under the Windows 10 operating system. MS-FINDER requires specially formatted MS^1^ and MS^2^ files as input. The settings are listed in Additional file 1: Table S1. The MS-FINDER program has a resource folder where two databases are located that the software uses to rank the candidate structures. The file ExistStructureDB_vs8.esd is an internal structure lookup database and the file ExistFormulaDB_vs8.efd (comprising formula from 13 metabolomics databases) is used to prioritize generated molecular formulas. These databases were emptied in order to evaluate the pure in silico fragmentation performances for challenge 2 and a new database was created, analogous to the one of MS-FINDER, using candidate structures provided by the CASMI organizers.

Both databases were opened in Notepad++ and all data except the header row was deleted and saved in the same format. Settings were adjusted to ±5 ppm mass accuracy and all compounds were processed in batch mode. Detailed information about the process can be obtained from the supplement section.

#### CFM-ID

The CFM-ID software (version 2.2, revision 26) was downloaded from https://sourceforge.net/projects/cfm-id/and was used on a server with 48-core AMD Opteron 6344 processor (2.6 GHz) running CentOS Linux 7. Out of several available command line utilities, the cfm-id executable was used for this project. Given an input spectrum and a list of candidate SMILES (or InChI) as provided by CASMI, cfm-id computes a predicted spectrum for each candidate and compares it to the input spectrum. It returns a ranking of the candidates according to how closely they match. The original CFM positive and negative models were used for the spectrum prediction, which were originally trained on data from the METLIN database. Mass tolerances of ±5 ppm were used and the Jaccard score and dot product score were applied for spectral comparisons. The dot product produced better rankings when applied in the voting/consensus model and was therefore used. The input spectrum was repeated for the low, medium and high energies, which originally emulate 10, 20 and 40 eV CID MS/MS spectra. Additional information is contained in Additional file 1.

#### MetFragCL

The command line version of MetFragCL software (version 2.2-CL) was downloaded from https://github.com/c-ruttkies/MetFrag and was used on MacBook Pro with 2.7 GHz Intel Core i5 and 16 GB DDR3. MetFragCL needs a parameter file of specific layout as input and it contains all necessary information for the processing of a given MS/MS peak list. Parameters for fragmentation are shown in Additional file 1: Table S2. Candidate files were prepared with the same application used for the analysis of the results, as mentioned previously. Finally, the in silico fragments are matched against the query peak list provided by CASMI. The measured peaks correspond to the charged fragments, so the matching function adds (positive mode) or removes (negative mode) a proton (1.007 Da) to or from the fragment mass. Additional settings are described in Additional file 1.

#### MAGMa+

The MAGMa+ software was downloaded from https://github.com/savantas/MAGMA-plus and was used on a cluster node with a 48-core AMD Opteron 6344 processor running CentOS Linux 7. MAGMa+ is an optimized version of the software MAGMa and is written as a Python wrapper script with identical command line arguments as the original MAGMa program with few changed parameters. Each candidate molecule was used to annotate the corresponding spectral tree with in silico generated substructures according to the algorithm published previously [15]. A Python script (process_hmdb.py) is provided that generates an SQLite.db database file from the public HMDB.sdf structures file, which is then used when running MAGMa. This script was modified to produce an analogous database file from the provided InChIs for each set of CASMI candidates. An additional Python script was written to generate spectral-tree files required by MAGMa from the CASMI peak lists and metadata. Additional information can be found in Additional file 1.

## Results

### CASMI Category 2 (Best Automatic Structural Identification: In Silico Fragmentation Only): parameter optimization and development of a voting/consensus model

**Fig. 1:**
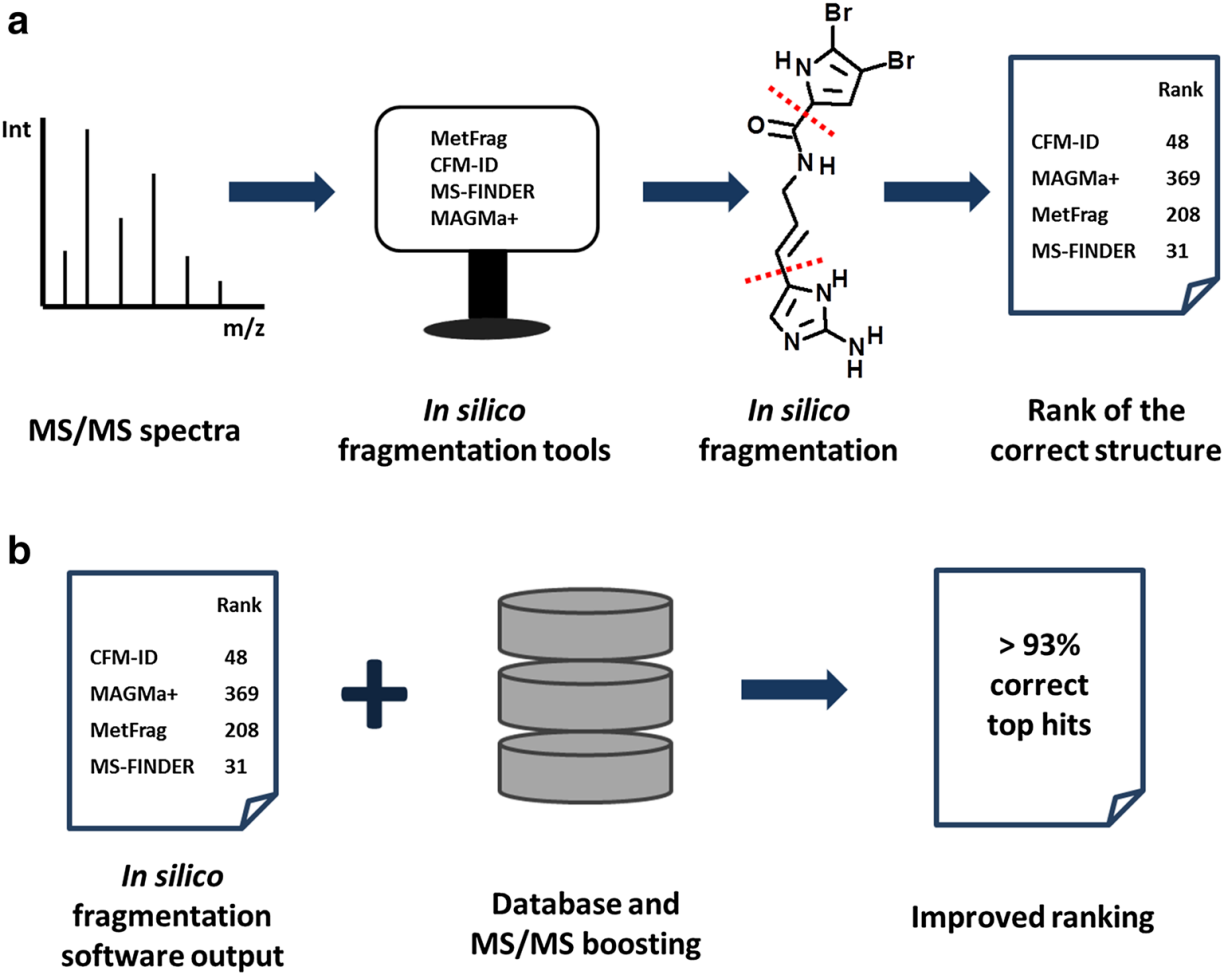
Structure elucidation workflow of small molecules. **a** In silico fragmentation can be used to identify and rank unknown MS/MS spectra by matching theoretical fragments to experimental MS/MS spectra. **b** The voting/consensus combines the output of multiple in silico fragmentation tools, uses compound and MS/MS databases lookups to further boost compound ranks

**Fig. 2:**
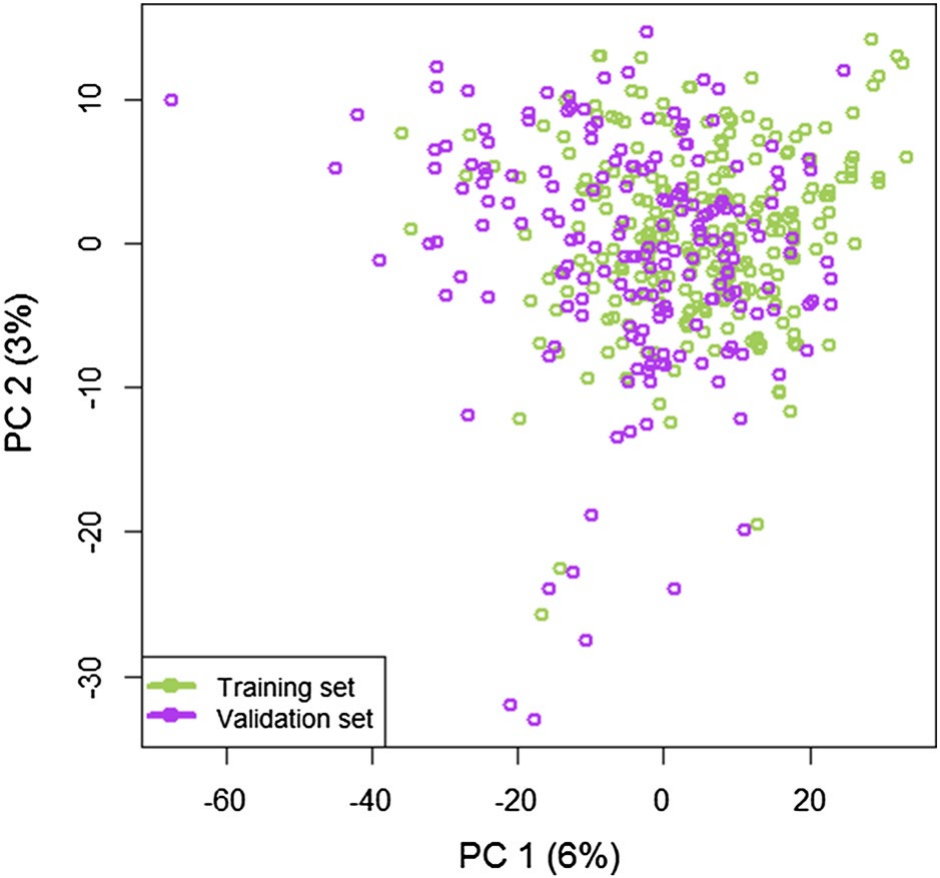
Principal component analysis of the molecular descriptor space from the training and validation sets. Individual outliers show compounds only found in a specific data set. Overlapping dots show very similar compounds

We tested the four tools that were used in the CASMI challenge, and for which the authors of the tools submitted result data by the CASMI deadline. Figure 1 gives the overview of our workflow for comparing results for CASMI Category 2 (in silico fragmentation tools only, Fig. 1a) and CASMI Category 3 (complementing results of in silico fragmentation tools with metadata queries, here: presence in chemical databases and MS/MS libraries). We first investigated whether the structures used in the CASMI training and validation sets were similar to each other. We decomposed the structures into molecular descriptors (structure fingerprints) and used these for variance analysis by Principal Component Analysis (PCA), (see Fig. 2). This analysis showed that both data sets were structurally highly similar, and only few compounds in the validation set were structurally different from the training set. Indeed, discrepancies in structure similarities between model building and model testing will certainly also occur when researchers try to identify unknown compounds in exposome or metabolomics research, as one cannot expect that ‘unknowns’ in untargeted profiling experiments will indeed fully resemble structures of identified compounds.

Not surprisingly, simple parameter optimization already resulted in improved structure annotation accuracies in comparison to the results the tool authors had submitted to CASMI. Such parameter optimization includes using a 5 ppm window for spectral comparison. Detailed parameter setting for each tool is listed in the Additional file 1. Secondly, each tool provided a ranked list of all MS/MS spectra (training and challenge) which was then used as an input for voting/consensus model resulting in new improved rankings, as described below.

#### In silico performance using the training set

Following the guidelines of the Category 2 challenge by the 2016 CASMI organizers, we evaluated each in silico software individually by using the best recommended settings and without secondary database rankings or use of other metadata. We utilized the 312 MS/MS spectra from the CASMI training set for parameter optimization of each tool and development of voting/consensus model. The number of compounds to be queried for each individual case ranged from less than 20 to over 8000 compounds. The individual software tools were able identify between 10 and 17% of the training set as top hits (see Table 1). CFM-ID ranked the correct metabolite first in 15% of the cases and 40% as top 5 candidates. MS-FINDER ranked the correct metabolite first in 10% of cases and 27% in the top 5. MAGMa+ ranked 16% of the compounds correctly. MetFragCL was the best performing tool in our comparison placing 17% cases correctly in the top rank and 43% in the top 5 hits (Table 1).

**Table 1:** Results for the training data of the CASMI 2016 contest

#	Tools	Top hits	Top 5	Top 10	Top 20
1	MetFrag + CFM-ID + DB + MS/MS Voting/consensus	290	304	305	306
2	CFM-ID + ID_sorted + MAGMa(+) + DB + MS/MS Voting/consensus	289	304	306	308
3	MetFrag + ID_sorted + DB + MS/MS Voting/consensus	288	305	306	308
4	MetFrag + DB + MS/MS	288	305	305	307
5	MAGMa(+) + ID_sorted + DB + MS/MS Voting/consensus	288	304	307	309
6	CFM-ID + ID_sorted + MAGMa(+) + MetFrag + DB + MS/MS Voting/consensus	288	304	305	308
7	MetFrag + CFM-ID + MAGMa(+) + DB + MS/MS Voting/consensus	288	304	305	307
8	CFM-ID + MAGMa(+) + DB + MS/MS Voting/consensus	288	303	306	307
9	MetFrag + MAGMa(+) + DB + MS/MS Voting/consensus	288	303	305	307
10	CFM-ID + ID_sorted + DB + MS/MS Voting/consensus	287	304	306	308
11	CFM-ID + DB + MS/MS	287	304	304	306
12	ID-sorted + DB + MS/MS	286	306	306	308
13	MetFrag + MS-FINDER + DB + MS/MS Voting/consensus	286	302	305	307
14	MS-FINDER + CFM-ID + DB + MS/MS Voting/consensus	286	301	304	305
15	MAGMa(+) + DB + MS/MS	286	301	302	303
16	MetFrag + MS-FINDER + CFM-ID + DB + MS/MS Voting/consensus	285	303	305	307
17	MS-FINDER + ID_sorted + DB + MS/MS Voting/consensus	285	302	306	307
18	MetFrag + MS-FINDER + CFM-ID + MAGMa(+) + DB + MS/MS Voting/consensus	285	302	305	307
19	MS-FINDER + DB + MS/MS	285	300	302	303
20	CFM-ID + ID_sorted + MAGMa(+) + MetFrag + MS-FINDER + DB + MS/MS Voting/consensus	284	303	306	307
21	MetFrag + MS-FINDER + MAGMa(+) + DB + MS/MS Voting/consensus	284	302	306	306
22	MS-FINDER + MAGMa(+) + DB + MS/MS Voting/consensus	284	301	305	306
23	MS-FINDER + CFM-ID + MAGMa(+) + DB + MS/MS Voting/consensus	283	302	305	305
24	MetFrag + CFM-ID + DB Voting/consensus	243	291	296	304
25	MetFrag + MS-FINDER + CFM-ID + DB Voting/consensus	242	289	298	301
26	MetFrag + CFM-ID + MAGMa(+) + DB Voting/consensus	240	290	297	304
27	MS-FINDER + DB	239	284	294	296
28	MetFrag + DB	238	290	296	301
29	MS-FINDER + CFM-ID + DB Voting/consensus	238	287	297	298
30	MS-FINDER + CFM-ID + MAGMa(+) + DB Voting/consensus	237	288	298	300
31	CFM-ID + MAGMa(+) + DB Voting/consensus	236	289	298	303
32	MetFrag + MS-FINDER + DB Voting/consensus	236	289	297	300
33	MetFrag + MS-FINDER + MAGMa(+) + DB Voting/consensus	236	288	298	300
34	MAGMa(+) + DB	236	287	294	299
35	CFM-ID + DB	236	286	295	302
36	MetFrag + MAGMa(+) + DB Voting/consensus	235	290	298	301
37	MS-FINDER + MAGMa(+) + DB Voting/consensus	235	288	298	299
38	ID-sorted + DB	227	291	301	303
39	Randomize + DB + MS/MS	195	273	289	305
40	Randomize + DB	193	268	283	298
41	ID-sorted	143	249	267	270
42	MetFrag + CFM-ID in silico Voting/consensus	69	155	194	230
43	MetFrag + CFM-ID + MAGMa(+) in silico Voting/consensus	62	154	187	228
44	MetFrag + MS-FINDER + CFM-ID + MAGMa(+) in silico Voting/consensus	62	145	180	228
45	MetFrag + MS-FINDER + CFM-ID in silico Voting/consensus	58	145	179	221
46	MS-FINDER + CFM-ID + MAGMa(+) in silico Voting/consensus	58	133	170	213
47	CFM-ID + MAGMa(+) in silico Voting/consensus	55	134	179	221
48	MetFrag in silico only	52	134	171	210
49	MetFrag + MAGMa(+) in silico Voting/consensus	52	133	171	210
50	MAGMa + in silico only	50	121	151	189
51	MS-FINDER + CFM-ID in silico Voting/consensus	50	111	141	188
52	MetFrag + MS-FINDER + MAGMa(+) in silico Voting/consensus	49	128	153	210
53	CFM-ID in silico only	48	124	170	209
54	MS-FINDER + MAGMa(+) in silico Voting/consensus	44	105	135	183
55	MetFrag + MS-FINDER in silico Voting/consensus	43	120	143	178
56	MS-FINDER in silico only	32	86	117	145
57	Randomize	4	13	27	46

‘MetFragCL, CFM-ID, MAGMa+ and MS-FINDER’ designate results obtained by the in silico fragmentation software tools. ‘DB’ designates priority ranking by presence in chemical and biochemical databases. ‘MS/MS’ designates presence in MS/MS libraries based on >400 dot-product similarity. 312 MS/MS spectra of the CASMI 2016 training data were used

#### Voting/consensus model

Each software provided candidate ranking for each MS/MS spectrum from the training and challenge data set. For ranking of the structures we considered only the first block of an InChiKey to discard enantiomeric or diastereomeric isomers. The voting/consensus model combines the ranking results of all tools and creates a new ranking system based on two criteria. The primary score of the voting/consensus model is calculated as the sum of the number of tools that successfully ranked a candidate compound. When all four tools found the candidate structure, this primary score was four. When none of the tools ranked a candidate, the score was zero. The secondary score for every voting/consensus model was calculated for each candidate structure by:

$$ S = \mathop \sum \limits_{A} \frac{{Ranking\;\left( {software\;A} \right)}}{{\omega \left( {top\;10\;software\;A} \right)}} $$

where ω represents the calculated sensitivity. The sensitivity for each software was calculated using a training data set as follows:

$$ \omega = \frac{correctly\;assigned\;structures}{correctly\;assigned\;structures + falsely\;assigned\;structures} $$

Correctly assigned structures were tested with different thresholds: top rank (the correct structure had to be ranked #1 by the software), top 5 (the correct structure had to be found within the top 5 structures), top 10 and top 20. We obtained best results when the sensitivity was calculated for the top 10 correctly assigned structures as shown in Table 3, and the calculated sensitivities were used for the validation set later on. By sorting the results in two levels with primary scores in descending and secondary scores in ascending order, new rankings are obtained for each candidate structure. The best voting/consensus model was chosen for each experiment. The voting/consensus model was written in R script and Java. The code is freely available at https://sourceforge.net/projects/insilico-fragmentation/. The application of the voting/consensus model to both categories is shown in the Fig. 1.

#### Voting/consensus model applied to in silico results

**Fig. 3:**
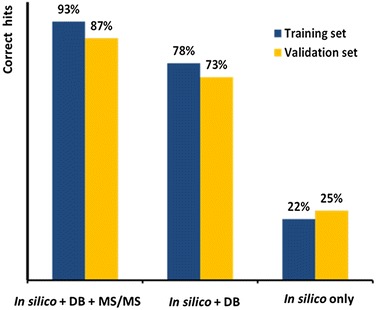
Comparison of the accuracy of compound annotations obtained by in silico fragmentation tools alone and in combination with metadata for both CASMI data sets

Subsequently we improved overall rankings by applying the voting/consensus model as detailed in the method section. In comparison to each individual tool’s results, the voting/consensus model built from a combination of MetFragCL and CFM-ID improved the overall results by 5%, ranking 22% cases in the top rank, 49% in the top 5 and 63% of the compounds in the top 10, an overview is shown in Fig. 3. The voting/consensus model takes into account the quality of each software which is why a secondary score is calculated using the number of hits in the top 10, assuming that experts will usually rely on and use the top 10 candidates proposed by a software.

### CASMI Category 3 (Best Automatic Structural Identification: Full Information): application of database and MS/MS similarity boosting

In Category 3, any additional information could be used to aid in the identification of the challenge spectra, for example, retention time information, mass spectral libraries, patents, reference count or biological relevance. Therefore, this CASMI category allowed a comparison of results obtained from pure in silico fragmentation tools with the integration of context metadata and in silico tools. Here, we exemplify the power of combining database presence with MS/MS similarity boosting in order to improve the accuracy of structure annotations from mass spectra, an approach which was successfully implemented previously [20].

Ultimately, a modified voting/consensus model was generated starting with the primary ranking obtained by the in silico fragmentation voting/consensus model sorted in descending order. The final score was then calculated by adding in the presence in compound databases, giving special emphasis on the presence of a structure in the STOFF-IDENT database, and adding MS/MS spectrum matching scores. When this ranking yielded a tie for two structures, the solution with the higher in silico ranking was given priority.

$$ Final\;score = in\;silico\;consensus\;rank + DB\;presence \,+\, 2XDB_{STOFF{-}IDENT} \,+\, 4XDB_{MS/MS} $$

The rationale for these boosting factors is given as follows:

(1) *Using database boosting* In silico fragmentation tools have never been published as a stand-alone tool without searching structure databases [21]. Querying public databases enables ranking in silico results according to the occurrence or importance of compounds. For example, if a candidate result structure is contained in multiple databases, it is most likely an often observed or important molecule, and might be more likely the correct structure than a less frequently observed isomer. Using this information would, hence, boost the ranking of isomers of in silico fragmentation tools. Other methods could employ the frequency of literature citations or the presence in target databases (e.g. for compounds known to be present in species or organs of interest). Here, we have used boosting structure rankings by its membership in the local database of MS-FINDER. This local compound database covers structures from the 13 most important metabolomic databases, including BMDB, CheBI, DrugBank, ECMDB, FoodDB, HMDB, KNApSack, PlantCyc, SMPDB, T3DB, UNPD, YMDB and STOFF-IDENT [22], containing 220,213 entries sorted according to InChIKey, PubChem, exact mass, formula and SMILES.

(2) *Using database presence emphasis factors* Many compounds in the CASMI training and challenge sets were environmentally relevant. We have therefore used the STOFF-IDENT compound database [22] which is used for environmentally relevant substances with a twofold boost factor. Other compound databases such as the EPA Dashboard [21] can be used accordingly. The boosting factor should be higher than one but lower than the MS/MS boosting factor. We recommend that this formula should be adapted when searching for structures that have other origins. When investigating endogenous metabolites, it is important to utilize biochemical databases like KEGG and to boost them accordingly.

(3) *Using mass spectral similarities* If an unknown compound has a perfect MS/MS similarity hit in a standard spectral library such as MassBank or NIST14, such a compound must be ranked very highly in the overall score. Even for medium spectral similarities, there are reasons to assume that differences between the experimental spectrum and library spectra might be due to differences in MS/MS parameters. Hence, candidate structures were boosted by mass spectral similarity matching against MS/MS libraries [23]. The NIST MS PepSearch program is a batch command-line version of the NIST MS search GUI program. Originally aimed at peptide scoring, this software can also be used for small molecule MS/MS similarity. Using.msp as input files, the NIST [24] and MassBank [11] MS/MS libraries were searched with a 5 ppm precursor window. Detailed parameters are listed in Additional file 1: Table S3. Out of 312 MS/MS spectra in the training set, 276 challenge spectra (88.4%) yielded hits in the MS/MS libraries with dot product scores ranging from very low similarity matches of 183 (for the training spectrum 109) up to optimal dot product scores of 999 (for the training spectrum 029). In the challenge data set, 208 MS/MS spectra were matched against the combined MassBank and NIST libraries. 125 spectra (60%) had positive matches with dot product scores ranging from 441 (for the challenge spectrum 182) up to a dot product score of 999 (for the challenge spectrum 049). We tested different cut offs for dot product matching scores in order to determine which threshold yielded results with most true positive compound annotations and the fewest false positive identifications. We found that for this CASMI data set, a dot product score threshold of 400 gave the best results on the training MS/MS spectra data set. We therefore used the same threshold for the CASMI challenge data set.

In order to ensure that good hits in the MS/MS spectral comparisons were given a high priority in ranking, we boosted hits for MS/MS similarity by a four-fold factor. We did not further use the actual MS/MS similarity match score but only the presence of an ‘MS/MS dot score >400’ hit, because the CASMI spectra represented data from different experiments and different MS/MS conditions, similar to spectra in NIST and MassBank libraries. The final rankings were obtained by sorting the sum of the scores in descending order. The higher the final score—the higher the new ranking.

Using this multi-parameter model, we boosted the overall accuracy of the model significantly by including each individual in silico fragmentation tool.

(4) *Using other metadata* We also noted that CASMI structure entries were listed by ChemSpider numbers, a database listing over 50 million chemicals [25]. ChemSpider entries are numbered by increasing numbers according to date of entry. We hypothesized that early ChemSpider entries (with low entry numbers) might be more relevant than high-entry numbers and tested if simple ID-number ranking (reported as ID-sorted) improved the overall ranking accuracy (Table 1).

A total of 57 different combinations were tested and the related data can be found in Table 1. The best voting/consensus model, built on CFM-ID and MetFragCL and it placed ~93% correctly in the top rank and ~98% in the top 10. However, it should be noted that simple boosting (i.e. querying for presence in databases or MS/MS similarity libraries) yielded almost as good results as a combination of in silico fragmentation and database/library boosting (Table 1): boosting alone yielded 286 correct hits which was only slightly worse than the best combination of in silico fragmentation tools and boosting. In comparison, in silico fragmentation tools alone (without boosting), yielded a maximum of 69 correct hits, even in a voting/consensus model, and a maximum of 52 correct hits when a single in silico tool was used.

#### Validation set performance for Categories 2 and 3

Finally, all 57 combinatorial methods were calculated on the training set and subsequently applied to the validation set (Table 2). This validation result mimics the approach an experienced investigator would take when identifying unknown compounds, by developing and tuning and cross-validating the algorithm on the training set and then applying the optimized parameters on the validation set. Again, each tool was used individually without any additional information and the voting/consensus model was applied using the weights calculated from the training set (see Table 3).

**Table 2:** Results for the challenge (validation) data of the CASMI 2016 contest

#	Tools	Top hits	Top 5	Top 10	Top 20
1	CFM-ID + ID_sorted + DB + MS/MS Voting/consensus	181	194	201	204
2	CFM-ID + ID_sorted + MAGMa(+) + DB + MS/MS Voting/consensus	180	195	200	205
3	CFM-ID + ID_sorted + MAGMa(+) + MetFrag + DB + MS/MS Voting/consensus	180	194	200	204
4	CFM-ID + DB + MS/MS	180	193	199	201
5	MAGMa(+) + ID_sorted + DB + MS/MS Voting/consensus	180	193	197	201
6	CFM-ID + MAGMa(+) + DB + MS/MS Voting/consensus	180	192	195	202
7	MetFrag + MAGMa(+) + DB + MS/MS Voting/consensus	180	188	194	198
8	MAGMa(+) + DB + MS/MS	180	188	192	198
9	MetFrag + CFM-ID + MAGMa(+) + DB + MS/MS Voting/consensus	179	190	196	201
10	MetFrag + CFM-ID + DB + MS/MS Voting/consensus	178	192	199	203
11	CFM-ID + ID_sorted + MAGMa(+) + MetFrag + MS-FINDER + DB + MS/MS Voting/consensus	175	191	200	203
12	MetFrag + MS-FINDER + CFM-ID + DB + MS/MS Voting/consensus	175	189	194	200
13	MetFrag + MS-FINDER + CFM-ID + MAGMa(+) + DB + MS/MS Voting/consensus	175	189	194	200
14	MS-FINDER + ID_sorted + DB + MS/MS Voting/consensus	175	189	194	199
15	MS-FINDER + CFM-ID + MAGMa(+) + DB + MS/MS Voting/consensus	175	188	196	201
16	MetFrag + MS-FINDER + MAGMa(+) + DB + MS/MS Voting/consensus	175	186	191	197
17	MS-FINDER + MAGMa(+) + DB + MS/MS Voting/consensus	175	185	190	195
18	ID_SORTED + DB + MS/MS	174	195	198	204
19	MetFrag + ID_sorted + DB + MS/MS Voting/consensus	174	194	199	203
20	MS-FINDER + CFM-ID + DB + MS/MS Voting/consensus	174	189	195	201
21	MetFrag + DB + MS/MS	174	189	192	197
22	MetFrag + MS-FINDER + DB + MS/MS Voting/consensus	174	187	190	197
23	MS-FINDER + DB + MS/MS	174	184	185	191
24	MetFrag + CFM-ID + MAGMa(+) + DB Voting/consensus	151	184	192	198
25	CFM-ID + DB	151	183	191	197
26	MetFrag + MS-FINDER + CFM-ID + DB Voting/consensus	151	180	191	198
27	MS-FINDER + CFM-ID + MAGMa(+) + DB Voting/consensus	151	179	191	198
28	CFM-ID + MAGMa(+) + DB Voting/consensus	150	184	189	199
29	MetFrag + MAGMa(+) + DB Voting/consensus	150	181	189	194
30	MetFrag + MS-FINDER + MAGMa(+) + DB Voting/consensus	150	178	186	193
31	MS-FINDER + MAGMa(+) + DB Voting/consensus	150	174	183	191
32	MetFrag + CFM-ID + DB Voting/consensus	149	186	196	201
33	MAGMa(+) + DB	149	180	185	193
34	MS-FINDER + CFM-ID + DB Voting/consensus	149	179	189	199
35	MS-FINDER + DB	148	173	178	186
36	MetFrag + DB	147	185	188	194
37	MetFrag + MS-FINDER + DB Voting/consensus	147	178	184	193
38	ID_SORTED + DB	134	188	194	202
39	Randomize + DB + MS/MS	123	184	189	197
40	Randomize + DB	119	176	180	189
41	ID_SORTED	106	169	177	186
42	MetFrag in silico	53	92	111	137
43	MetFrag + MS-FINDER + CFM-ID in silico Voting/consensus	51	95	129	151
44	MetFrag + CFM-ID in silico Voting/consensus	47	102	129	153
45	MetFrag + MS-FINDER + CFM-ID + MAGMa(+) in silico Voting/consensus	46	97	128	152
46	MetFrag + CFM-ID + MAGMa(+) in silico Voting/consensus	42	104	126	150
47	CFM-ID + MAGMa(+) in silico Voting/consensus	39	94	123	148
48	MetFrag + MAGMa(+) in silico Voting/consensus	39	90	111	128
49	MetFrag + MS-FINDER + MAGMa(+) in silico Voting/consensus	38	79	117	138
50	MS-FINDER + CFM-ID + MAGMa(+) in silico Voting/consensus	34	97	127	147
51	MetFrag + MS-FINDER in silico Voting/consensus	33	76	103	125
52	MS-FINDER + MAGMa(+) in silico Voting/consensus	32	69	93	119
53	MS-FINDER + CFM-ID in silico Voting/consensus	30	76	110	139
54	CFM-ID in silico (dot product)	29	76	104	122
55	MAGMa(+) in silico	28	72	98	117
56	MS-FINDER in silico	23	57	79	93
57	Randomize	20	27	28	121

‘MetFragCL, CFM-ID, MAGMa+ and MS-FINDER’ designate results obtained by the in silico fragmentation software tools. ‘DB’ designates priority ranking by presence in chemical and biochemical databases. ‘MS/MS’ designates presence in MS/MS libraries based on >400 dot-product similarity. 208 MS/MS spectra of the CASMI 2016 training data were used

**Table 3:** Sensitivity, ω, calculated for each tool (MetFragCL, CFM-ID, MAGMa+ and MS-FINDER) based on the correctly assigned structures in the top rank, top 5, top 10 and top 20 using the training data set of 312 MS/MS spectra

#	Tools	Top hits	Top 5	Top 10	Top 20
1	MetFragCL in silico only	0.1666	0.4294	0.548	0.673
2	MAGMa+ in silico only	0.1602	0.3878	0.4839	0.6057
3	CFM-ID in silico only	0.1538	0.3974	0.5448	0.6698
4	MS-FINDER in silico only	0.10256	0.27564	0.3750	0.4647

The sensitivity was calculated as follows: ω = true positive/(true positive + false negative). The calculated sensitivities were used on the challenge data set

The validation set corroborated the findings from the training set performances. With 25% correctly assigned structures as the top hit, MetFragCL was the best stand-alone in silico fragmentation tool. CFM-ID followed with 14% correctly identified compounds and MAGMa+ and MS-FINDER identified less than 14% correct. The voting/consensus model built on MetFragCL, CFM-ID and MS-FINDER did not improve the top hit results of MetFrag, however there were 9% more correctly assigned structures noted in the top 10.

When boosting the pure in silico outputs by database presence and MS/MS scoring, the best individual tool to use was CFM-ID, correctly assigning 86% of the cases in the top rank. Indeed, results for each of the in silico tools were drastically improved by DB and MS/MS boosting. The best results (top hit) were obtained with 87% correct annotations for the CFM-ID and ID-sorted voting/consensus model. Additional file 2 contains all the combinatorial methods that were used but were not shown in the manuscript.

#### Calculation times

We investigated a total of 520 compounds. However, each individual in silico tool had to process 691,319 compounds from the query database. This large number of database compounds made it challenging for a number of tools. Performance-wise, MetFragCL was the fastest with only a 12-h calculation time for the 312 training compounds, MAGMa+ needed 18 h, whereas MS-FINDER needed one day, using a regular personal computer as given in the method section. CFM-ID needed two days on a 48-CPU cluster to finish the calculation of the training set. Here additional time-out parameters can be set in the future to avoid very long computational times for individual compounds.

## Discussion

Results uploaded to the CASMI contest website as well as our post hoc tool comparison clearly show that in silico algorithms alone are still far away from practical use for identification of true unknowns, for example, compounds that are not currently represented in chemical or biochemical databases. Only 17% of the answers were correctly annotated structures from MS/MS spectral information in the training data set. Even when combining all in silico tools in a voting/consensus model, only 22% of the compounds were ranked as top-candidates. Importantly, even these numbers rest on the assumption that ‘unknowns’ detected in LC–MS/MS of metabolomic or environmental studies are present as existing structures in PubChem or ChemSpider, as CASMI gave lists of potential structures that were to be ranked using ChemSpider structures. It is very likely that in actual untargeted profiling studies, many structures would have to be considered ‘unknown unknowns’, i.e. compounds that have not been structurally described before and that are not represented by the 70 million compounds found in PubChem or ChemSpider. Few approaches exist to enumerate such database derivative structures, for example the ‘metabolic in silico network expansion DB (MINE)’ [23]. Completely de-novo spectra-to-structure calculations are yet impossible.

Currently, best results were obtained when structure database importance and MS/MS search were used along with in silico voting/consensus models. Interestingly, each of the in silico tools experienced tremendous boosts, leading to 93% correctly assigned structures when combining CFM-ID and MetFragCL results in the training data set. Indeed, combined approaches have been successfully used in past CASMI challenges [6, 12, 26, 27]. However, previous challenges did not include a large enough number of compounds for full testing. Our step-wise combinatorial multi-model approach shows a more detailed view of overall performances. Once customizable tools are available, we will extend our searches to other in silico fragmentation algorithms such as CSI:FingerID [9] or the novel Input Output Kernel Regression models (IOKR) [28]. The two latter tools are of interest, because they are user-friendly, very fast and were top performers in the official CASMI contest in the in silico-only category. CSI:FingerID currently does not allow for localized database search, and CSI:IOKR is still not publicly available.

The compounds provided in the CASMI 2016 contest were environmental xenobiotics and drugs, all covered in structure databases. About 70 MS/MS spectra had not yet been deposited in commercial or publicly available MS/MS databases. Therefore, these MS/MS spectra were not available for any software to be used as training sets, rendering these spectra an excellent test for the CASMI 2016 contest to test in silico fragmentation algorithms. Moreover, 37,957 compounds contained fluorine atoms in their structure, for which fragmentation patterns are harder to interpret. Similar to real LC–MS/MS runs, a range of challenge MS/MS spectra were sparse in the number of product ion peaks, causing in silico tools to fail for lack of data (Additional file 1: Table S4). While often hundreds of isomers are retrieved per chemical formula, annotation tools must fail if too few MS/MS product ions are generated [29]. We recommend acquiring and combining MS/MS data under multiple collision energies, or even with different mass spectrometers, for important unknowns that are detected as statistically significant in metabolomics studies.

Offering a command-line version for in silico fragmentation software that is capable to run batches of tests is required to process potentially thousands of unknown tandem mass spectra from profiling studies. Multi-threading and use of all CPU cores is required. However, the true challenge lies in providing tools that can be used in batch mode, but are still user-friendly enough for untrained investigators. Many of these software tools operate across Windows, Linux and MacOS and require different libraries and dependencies, demanding a team environment that is skilled in cheminformatics techniques. Structure clean-up steps from the provided structure databases proved to be time-consuming, involving tasks such as removing counter ions, adduct salts, or isotopes. Offering a web-based research tool is recommended. However, data transfer over the web is often forbidden in industrial environments and is also prone to network errors and server outages. Because almost all mass spectrometry vendors use the Windows platform, we further recommend providing Windows-based tools for in silico fragmentations. For individual performance checking it is also useful to investigate each individual result with graphical user interfaces. Here MS-FINDER provides a convenient desktop solution for Windows.

Our newly developed voting/consensus model software can automatically evaluate hundreds of optimization models and report overall outcomes or top hits, top ten hits and the specificity of the model. Our software is suited to be extended to include more in silico software tools, with output statistics to be modified to calculate additional statistical figures of merit. Such extensions require that in silico software tools are publicly available in a usable form so the results can be independently validated. One could imagine future CASMI contests completely run automatically by software, preventing errors and individual interventions.

## Conclusions

In silico algorithms for structural fragmentation of compounds are still in early development. In many cases, existing tools only cover simple fragmentations, but not more complex rearrangement reactions [29]. Once more MS/MS spectra become available and corresponding structural diversity increases, these can be used to train and optimize in silico algorithms which will lead to better performance [30].

Pure in silico algorithms only identified 17–25% of the compounds correctly. Once the database and MS/MS search were added, the algorithm was able to correctly identify 87–93% of the “known-unknown” compounds as the first hit. Our results show that for the “known-unknown” compounds the choice of in silico fragmentation software is negligible, when database and MS/MS boosting are aiding the annotation process. These results confirm that voting/consensus models can be used for real-world applications. Our software will also allow for automatic testing and performance tuning without user interaction.

The true challenge is presented by the identification of “unknown unknown” compounds that are not yet covered in compound databases or that are computationally derived as chemical or enzymatic derivatives [23]. Here classical experimental ways of structure elucidation, including compound purification and subsequent NMR, UV and MS will play a role in elucidating the correct isomer structure.

## Additional files

**Additional file 1.** Contains detailed parameter settings for each tool used in this research.

**Additional file 2.** Contains results of all calculations and combinations of the tools that were used but were not shown in the manuscript.
