# Supplementary material for: Comprehensive comparison of in silico MS/MS fragmentation tools of the CASMI contest: database boosting is needed to achieve 93% accuracy
Source: J Cheminform. 2017 May 25;9:32. doi: 10.1186/s13321-017-0219-x (PMC5445034; doi:10.1186/s13321-017-0219-x)
Supplement: Supplementary file 1 — Additional file 1. Contains detailed parameter settings for each tool used in this research. [file 13321_2017_219_MOESM1_ESM.docx]

# Comprehensive comparison of in silico MS/MS fragmentation tools of the CASMI contest: database boosting is needed to achieve 93% accuracy

Ivana Blaženović ^1,2,3^, Tobias Kind ^3^, Hrvoje Torbašinović ^4^, Slobodan Obrenović ^4^, Sajjan S. Mehta ^3^, Hiroshi Tsugawa ^5^, Tobias Wermuth ^3^, Nicolas Schauer ^2^, Martina Jahn ^1^, Rebekka Biedendieck ^1^, Dieter Jahn ^1^, Oliver Fiehn ^3,6§^

^1^ Technische Universität Braunschweig · Institute of Microbiology, Braunschweig, Germany

^2^ Metabolomic Discoveries GmbH, Potsdam, Germany

^3^ West Coast Metabolomics Center, UC Davis, Davis, CA, U.S.A.

^4^ Inovatus Ltd, Zagreb, Croatia

^5^ RIKEN Center for Sustainable Resource Science, Yokohama, Kanagawa, Japan.

^6^ Department of Biochemistry, Faculty of Sciences, King Abdulaziz University, Jeddah, Saudi‑Arabia

^§^Corresponding author

Prof. Oliver Fiehn, PhD

Director, NIH West Coast Metabolomics Center

UC Davis Genome Center, room 1313

451 Health Sci Drive

Davis, CA 95616

office phone +1-530-754-8258

lab phone +1-530-752-9922

cell phone +1-530-723-4450

# Methods

The CASMI 2016 website (<http://www.casmi-contest.org/2016/>) provided files contained MS/MS information as *.MGF file as well as structures for each of the challenge and validation sets. That included the ChemSpider ID, compound name, the monoisotopic mass, the molecular formula, SMILES, InChI and InChIKey.

Tools that were used for this research, information how to install and use them are presented below.

**Software settings**

**MS-FINDER**

MS-FINDER software and its documentation was taken from its website <http://prime.psc.riken.jp/Metabolomics_Software/MS-FINDER/index.html>.

MS-FINDER program has a resources folder where two databases are located and that the software uses to rank the candidate structures (ExistStructureDB_vs8.esd) and to generate molecular formulas (ExistFormulaDB_vs8.efd). These databases have to be emptied in order to evaluate the pure in-silico fragmentation performances. Under normal circumstances this is not required. Both databases were opened in Notepad++ and everything except the header row was deleted and saved in the same format. Unknown structures were imported as user-defined db by merging all candidates .csv files provided by CASMI using the following steps: a) Windows Start Button. b) Type cmd and hit enter ("Command Prompt" in Windows 10). c) Go to the folder with the CSV files (for help how to do that enter "help cd"). d) Type copy *.csv all.txt and hit enter to copy all data in the files into all.txt. e) Type exit and hit enter to close the DOS window. f) Open Excel. g) Click File Open to open all.txt file that was saved in the same folder where the candidate .csv files are. h) Choose Delimited. i) Next. j) Check Comma. k) Finish. l) Remove all the headers from newly created file and change the structure of the table according to the file located in the MS-FINDER Local DB example folder. m) Save as .txt file. Now, user-defined database is ready to use. Once all the settings were applied, databases and MS/MS data have been prepared, in the Analysis tab Compound annotation (batch job) was selected followed by Batch job settings set to both processes (formula finder and then structure finder) and top N hits was set to 12176 for the training and to 8555 for the challenge set (this corresponds to the candidate files with the highest number of possible structures provided by CASMI, to ensure that all given structures are considered and ranked by the software).

**MetFragCL**

Command line version of the tool and its documentation was downloaded from its website and used on a Mackintosh <http://c-ruttkies.github.io/MetFrag/projects/metfragcl/>. The settings that were used to run the analysis for both sets, training and validation are listed in the Supplemental Table 2.

**MAGMa+:**

A Python script (process_hmdb.py) is provided that generates an SQLite .db database file from the public HMDB .sdf structures file, which is then used when running MAGMa. This script was modified to produce an analogous database file from the provided InChIs for each set of CASMI candidates. An additional Python script was written to generate spectral-tree files required by MAGMa from the CASMI peaklists and metadata. The docker images and associated docker files that were used with detailed settings can be found here <https://hub.docker.com/r/ssmehta/magma-plus/>. Command line to start the run is: docker run -it -v /path/to/magma-plus/supplement:/data ssmehta/magma-plus:v1.0.0.

Once the 4 .sdf files were generated, one for positive and negative mode as well as for training and challenge data sets, they were now ready to be converted into MAGMA+ structure database file. MAGMa+ parameters that we used include -i (ionization mode: -1 for negative and 1 for positive mode), -p (maximum relative ppm error), -q (maximum absolute m/z error in Da), -c (minimum intensity of MS1 precursor ion peaks to be annotated), -d (minimum intensity of fragment peaks to be annotated, as percentage of base peak) and. The following settings were applied: read_ms_data -i -1 /1, -p 10 , -q 0.01, -c 0, -d 0 and -s hmdb.

**CFM-ID**

The original CFM positive and negative models were used for the spectrum prediction, which were trained on data from the Metlin database. Mass tolerances of 5 ppm were used and the Jaccard score and DotProduct score were applied for spectral comparisons, the better rankings produced by this comparative method were used for final evaluation. The input spectrum was repeated for the low, medium and high energies, which originally emulates 10, 20, 40 eV CID, however this information was not available. The docker images and associated docker files that were used and the detailed settings can be found here <https://hub.docker.com/r/ssmehta/cfmid/> and the command line to run the tool and reproduce all the results is: docker run -it -v /path/to/cfmid/supplement:/data ssmehta/cfmid:r25.

**MS/MS database search**

The NIST MS PepSearch program is a batch command-line version that is related to the NIST MS Search GUI program. The input files were .msp files and the NIST and MassBank MS/MS libraries were searched with a 5 ppm precursor window. Detailed parameters are listed in Supplemental Table 3.

**CSI: FingerID**

CSI: FingerID combines computation and comparison of fragmentation trees with kernel methods. The kernel denotes a similarity coefficient for either MS/MS spectra or fragmentation trees and is used for the prediction of molecular properties of the unknown compound. Currently it is not possible to modify the candidate database locally thus we did not compare CSI: FingerID with the other tools in the main manuscript. However, it is of interest to see how it performs on the same data set we have incorporated the results in the supplement. CSI: FingerID was downloaded after the candidate structures were revealed and final results were published. The program is a 64 bit GUI version from <https://bio.informatik.uni-jena.de/software/sirius/> and was used on Windows. Settings are listed in the Supplemental Table 5. After the exclusion of 97 compounds that were marked as training set a total of 67 compounds (47.86%) were found correctly as a top hit. With inclusion of all the known compounds CSI:Finger ID was able to correctly annotate 140 MS/MS spectra of the validation set or 67,30%. However, since it was not possible to customize the local database in order to test the in silico fragmentation possibilities we did not compare these results to other tools.

# Authors' contributions

IB, TK, OF designed the experiment. IB and SM performed the analysis, IB and TK evaluated the results, TK interpreted the results, HT provided support with programming, SO designed the voting/consensus model. IB, TK, DJ, MJ, RB, NS and OF wrote the manuscript. All authors read and approved the final manuscript.

# Acknowledgements

Funding for T.K. and O.F. was supported by NSF MCB 1139644, NIH P20 HL113452 and U24 DK097154.  Additional funding for T.K. was provided by American Heart Association 15SDG25760020 (Irvin) and NIH 7R01HL091357-06 (Arnett). Furthermore, we are thankful to Deutsche Forschungsgemeinschaft (German Research Foundation), Bundesministerium für Bildung und Forschung (BMBF, the Federal Ministry for Education and Research) and we are grateful to the Cusanuswerk (KAAD) for support. We would like to thank CASMI 2016 participants for sharing their methods and parameter settings with us for this research: Felicity Allen, Christoph Ruttkies, Dries Verdegem and Arpana Vaniya. We are thankful to Boris Šlogar for revision and linguistic editing efforts.

# Tables

**Table S1** – Settings used for open source in silico fragmentation tool MS-FINDER

| **#** | **Settings** | **YES/NO or other information** |
| --- | --- | --- |
| 1 | Formula finder: Lewis and Senior check | yes |
| 2 | Isotopic ratio tolerance | 20% |
| 3 | Element ratio check | Common range (99.7%) covering |
| 4 | Element probability check | yes |
| 5 | Mass tolerance type | ppm |
| 6 | Mass tolerance (MS1) | 5 ppm |
| 7 | Mass tolerance (MS2): | 10 ppm |
| 8 | Element section (O, N, P, S, F, Cl, Br, I, Si): | yes |
| 9 | Result cut off | 12176 for training and 8555 for challenge data set. |
| 10 | Structure finder: In silico MS/MS fragmenter setting: Tree depth | 2 |
| 11 | Relative abundance cut off | 1% |
| 12 | Result cut off | 12176 for training and 8555 for challenge data set. |
| 13 | Local Databases | User-defined db |
| 14 | MINE database | Never use it |
| 15 | PubChem online setting | Never use it |

## Table S2 – Settings used for open source in silico fragmentation tool MetFragCL

| **#** | **Settings** | **Information** |
| --- | --- | --- |
| 1 | PrecursorIonMode | 1 (1 for positive and -1 for negative ionization mode) |
| 2 | IsPositiveIonMode | True (False for negative ionization mode) |
| 3 | FragmentPeakMatchAbsoluteMassDeviation | 0.001 |
| 4 | FragmentPeakMatchRelativeMassDeviation | 5 |
| 5 | MaximumTreeDepth | 2 |
| 6 | MetFragPostProcessingCandidateFilter | InChIKeyFilter |
| 7 | Adduct type of the precursor | [M+H ]+ for positive and [M-H]- for negative ionization mode |

**Table S3 –** Detailed parameters used for MS/MS search using the NIST MS PepSearch program

| **#** | **Settings** | **Information** |
| --- | --- | --- |
| 1 | Presearch mode | standard |
| 2 | m/z limits | Min. = 0 and Max. = 2000 |
| 3 | Search tolerance settings | Precursor ion tolerance, m/z units 0.005 |
| 4 | Ignore peaks around precursor | yes |
| 5 | Fragment peak m/z tolerance | 0.5 |
| 6 | Min. match factor (MF) to output (0-999) | 1 |
| 7 | Min. peak intensity (1-999) | 1 |
| 8 | Max. number of output hits | 1 |
| 9 | Show spectra without matches | yes |
| 10 | Include Hit-Unknown precursor m/z difference | yes |
| 11 | Include m/z in the output | yes |
| 12 | Output the input spectrum number | yes |
| 13 | Set program priority above normal | yes |
| 14 | Use number of replicates | no |
| 15 | Calculate rev-dot | yes |
| 16 | Q-TOF | no |
| 17 | Load libraries in memory | no |

**Table S4** – List of the compounds where every tool performed poorly. Ranking position calculated by each tool per correct solution is shown.

| **Correct solution** | **Ranking position of the correct compound given by in silico fragmentation tool** | | | |
| --- | --- | --- | --- | --- |
| **InChIKey (1^st^ block)** | **MetFragCL** | **MS-FINDER** | **CFM-ID** | **MAGMa+** |
| ZZORFUFYDOWNEF | 137 | 541 | 75 | 308 |
| YHQDZJICGQWFHK | 98 | 184 | 205 | 119 |
| BWHOZHOGCMHOBV | 119 | 182 | 125 | 156 |
| SYELZBGXAIXKHU | 234 | 105 | 85 | 144 |
| IRCMYGHHKLLGHV | 151 | 1161 | 181 | 460 |
| LCGTWRLJTMHIQZ | 123 | 111 | 124 | 129 |
| RJGDLRCDCYRQOQ | 169 | 186 | 57 | 210 |
| AFYCEAFSNDLKSX | 123 | 366 | 142 | 227 |
| VHBFFQKBGNRLFZ | 213 | 118 | 53 | 212 |
| CWJSHJJYOPWUGX | 576 | 24 | 125 | 341 |
| WWYNJERNGUHSAO | 113 | 71 | 73 | 193 |
| VVBLNCFGVYUYGU | 1031 | 5460 | 644 | 1825 |
| FRQMUZJSZHZSGN | 177 | 116 | 51 | 271 |
| MMBILEWCGWTAOV | 361 | 253 | 45 | 281 |
| CHIFOSRWCNZCFN | 79 | 675 | 1160 | 1238 |

**Table S5 –** Parameter setting for in silico fragmentation tool CSI:FingerID

| **#** | **Settings** | **Information** |
| --- | --- | --- |
| 1 | File type | .mgf |
| 2 | Elements beside CHNOPS | Br, Cl, F, I |
| 3 | Search tolerance settings | Precursor ion tolerance, m/z units 0.005 |
| 4 | Ionization | [M-H]- and [M+H]+ |
| 5 | Instrument | Q-TOF |
| 6 | ppm | 5 |
| 7 | Consider | PubChem formulas |
| 8 | Search with CSI:FingerID | PubChem |
| 9 | Export | CSI:FingerID results |

**Table S6 –** Comparison of the results submitted to CASMI contest versus results obtained in our research for the Category 2 – in silico only.

| **#** | **Tools** | **Original submission (top hit)** | **Our research** |
| --- | --- | --- | --- |
| 1 | MetFrag | 24 | 53 |
| 2a | CFM-ID (Jaccard) | 39 | 37 |
| 2b | CFM-ID  (dot product) | - | 29 |
| 3 | MAGMa+ | 24 | 28 |
| 4 | MS-FINDER | 46 | 22 |

**Table S7 –** Comparison of the results submitted to CASMI contest versus results obtained in our research for the Category 3 – everything allowed.

| **#** | **Tools** | **Original submission (top hit)** | **Our research** |
| --- | --- | --- | --- |
| 1 | MetFrag | 162 | 174 |
| 2 | CFM-ID | 117 | 180 |
| 3 | MAGMa+ | Did not participate | Did not participate |
| 4 | MS-FINDER | 146 | 174 |
